# Evaluating immunotherapeutic outcomes in triple-negative breast cancer with a cholesterol radiotracer in mice

**DOI:** 10.1172/jci.insight.175320

**Published:** 2024-03-19

**Authors:** Nicholas G. Ciavattone, Nan Guan, Alex Farfel, Jenelle Stauff, Timothy Desmond, Benjamin L. Viglianti, Peter J.H. Scott, Allen F. Brooks, Gary D. Luker

**Affiliations:** 1 Department of Radiology, and; 2 Department of Biomedical Engineering, University of Michigan, Ann Arbor, Michigan, USA.

**Keywords:** Immunology, Oncology, Breast cancer, Diagnostic imaging, Immunotherapy

## Abstract

Evaluating the response to immune checkpoint inhibitors (ICIs) remains an unmet challenge in triple-negative breast cancer (TNBC). The requirement for cholesterol in the activation and function of T cells led us to hypothesize that quantifying cellular accumulation of this molecule could distinguish successful from ineffective checkpoint immunotherapy. To analyze accumulation of cholesterol by T cells in the immune microenvironment of breast cancer, we leveraged the PET radiotracer, eFNP-59. eFNP-59 is an analog of cholesterol that our group validated as an imaging biomarker for cholesterol uptake in preclinical models and initial human studies. In immunocompetent mouse models of TNBC, we found that elevated uptake of exogenous labeled cholesterol analogs functions as a marker for T cell activation. When comparing ICI-responsive and -nonresponsive tumors directly, uptake of fluorescent cholesterol and eFNP-59 increased in T cells from ICI-responsive tumors. We discovered that accumulation of cholesterol by T cells increased in ICI-responding tumors that received anti–PD-1 checkpoint immunotherapy. In patients with TNBC, tumors containing cycling T cells had features of cholesterol uptake and trafficking within those populations. These results suggest that uptake of exogenous cholesterol analogs by tumor-infiltrating T cells allows detection of T cell activation and has potential to assess the success of ICI therapy.

## Abstract 2

Uptake of a cholesterol radiotracer in T cells correlates directly with activation and efficacy of anti-PD-1 therapy in mice with triple negative breast cancer

## Introduction

Immune checkpoint inhibitor (ICI) antibodies that block the PD-1 or CTLA-4 pathway have revolutionized cancer therapy. By preventing signals that turn “off” killing functions of T cells, ICIs can unleash host immunity against cancer cells, producing remarkable survival advantages and even cures for a minority of patients. Unfortunately, most patients do not benefit from ICIs, and some patients experience severe side effects of these drugs. Extending ICIs to breast cancer and other, less immunogenic tumors remains a substantial barrier in cancer immunotherapy. Recently, the anti–PD-1 ICI pembrolizumab received approval for treatment of high-risk, early-stage triple-negative breast cancer (TNBC) in July, 2021 (1). TNBC tumors, the least common and most aggressive subtype, typically show more inflammation than other subtypes of breast cancer. An inflamed, immune “hot” environment correlates with better outcomes in TNBC (2). Unfortunately, most patients with TNBC do not benefit from immunotherapy based on current metrics for selection and/or do not respond to ICIs (3). For example, in the KEYNOTE-355 trial, pembrolizumab significantly extended survival in patients categorized as favorable for immunotherapy based on high expression of PD-L1 in tumor cells, lymphocytes, and macrophages in biopsy samples (4). Despite improved overall survival, anti–PD-1 treatment allowed only 11% more patients to survive through the 4.5-year study endpoint relative to those treated only with chemotherapy. These data highlight shortcomings of existing criteria to evaluate patients likely to respond to immunotherapy and assess effects of treatment in breast cancer. Creating new, optimally noninvasive methods to assess immunotherapy in patients with TNBC and identify early effects of therapy on T cells will help optimize use of existing drugs and facilitate translation of new immunotherapies. Since only a minority of patients across all malignancies respond to current ICIs, better methods to select patients and monitor therapy likely will improve outcomes across multiple cancers.

Activated T cells are one of the hallmarks of an inflamed, effective antitumor immune response. Previous studies suggest a link between activation of T cells and cholesterol. T cells increase cholesterol through de novo synthesis from acetyl CoA or uptake from lipoproteins through receptor-mediated endocytosis of low-density lipoprotein (LDL) particles from the bloodstream. Cholesterol is an essential component of cellular membranes, where this lipid modulates membrane fluidity, permeability, and receptor-mediated signaling (5). Upon activation, T cell receptor (TCR) nanoclusters form in cholesterol-rich membrane domains to elicit strong TCR signaling (6). Proliferating T cells and other cell types require increased amounts of cholesterol to replicate cell membranes, utilizing cholesterol stored as cholesterol esters in intracellular lipid droplets. Despite requirements for cholesterol in T cell activation and proliferation, increased cholesterol uptake by tumor-infiltrating T cells also has been linked to an exhausted T cell phenotype (7). However, exhausted T cells indicate a history of active T cell populations, and both populations of T cells correlate with success of ICIs in TNBC (8, 9). Therefore, cholesterol uptake by tumor-infiltrating lymphocytes (TILs) could indicate a hot tumor environment and determine the efficacy of immunotherapy.

Our research group previously developed the fluorinated radiotracer, eFNP-59, as an analog for cholesterol that can be leveraged to detect cholesterol uptake by cells and tissues using positron emission tomography (PET) (10). eFNP-59 is a further improved acetate ester of FNP-59 that improves uptake compared with previous generations of this radiotracer (11). The fluorine-18–synthesized radiotracer overcomes limitations of previous iodine-131-NP59 that was used clinically to detect adrenal cortical tumors and maintains sensitivity to detect uptake in tissues and cells, including cells of the immune system (10). As a cholesterol analog, eFNP-59 distributes into the lipid pool and enters lipoproteins with subsequent trafficking into cells through receptors like the LDL receptor (LDLR) and fatty acid transporter, CD36. The fluorine-18 radiolabel is used commonly in radiotracers for PET imaging studies, such as the glucose analog 2-(^18^F) fluoro-2-deoxy-D-glucose (FDG) to detect glycolytic metabolism in cancer and other diseases (12, 13). We hypothesized that activated and primed T cells in the tumor microenvironment have increased uptake of cholesterol detectable with eFNP-59 as a marker for success of ICI therapy given cholesterol’s role.

This study reveals that increased uptake of cholesterol marks activation of T cells in mouse TNBC tumors responsive to ICI therapy, and we can distinguish T cells from ICI-responsive and -nonresponsive tumors with eFNP-59. Ex vivo restimulation of T cells recovered from ICI-responsive tumors in mice exhibited greater cholesterol uptake compared with nonresponsive tumors. In vivo, we discovered that T cells from responsive tumors accumulated significantly more cholesterol than nonresponsive tumors, the latter of which exhibited almost undetectable uptake. Lastly, data mining studies of human TNBC showed upregulation of genes related to uptake and trafficking of cholesterol in populations of activated T cells. These data demonstrate that uptake of cholesterol as measured with eFNP-59 demarks proliferating T cell populations and correlates with success of immunotherapy in preclinical mouse models of TNBC. Moreover, our work points to eFNP-59 as a potential radiotracer to improve monitoring of cancer immunotherapy.

## Results

### T cells from ICI-responsive tumors show greater cholesterol uptake.

**Figure 1:**
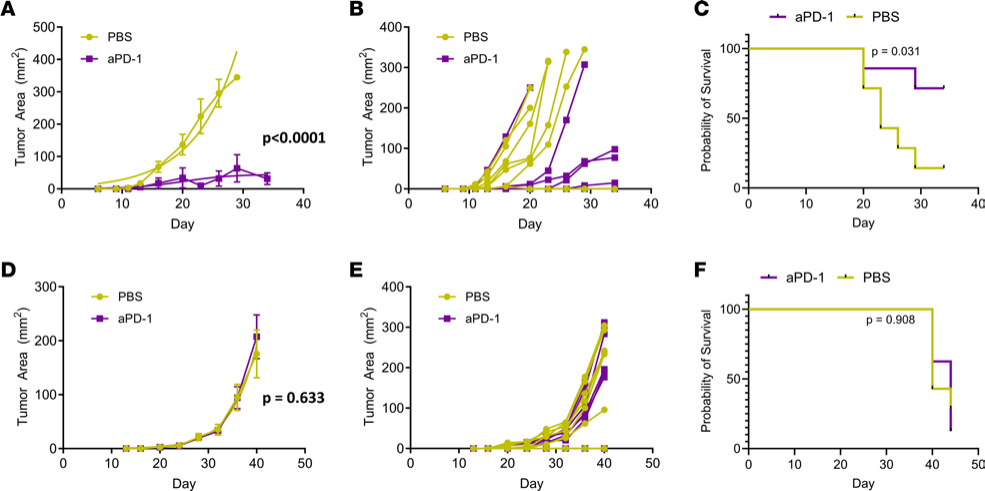
E0771 tumors respond to anti–PD-1 immunotherapy, while AT-3 tumors do not. Three days after orthotopically injecting E0771 or AT-3 breast cancer cells plus mouse mammary fibroblasts into syngeneic C57BL/6J mice, we randomly assigned animals to treatment with anti–PD-1 antibody or PBS vehicle every 3 days for 4 doses total. Graphs show mean values ± SEM (symbols) and calculated logistic regression (smooth line) for E0771 (**A**) or AT-3 (**D**) tumors (*n* = 6 control; *n* = 8 anti–PD-1) treated with anti–PD-1 antibody or PBS. (**B** and **E**) Growth of E0771 and AT3 tumor growth, respectively, for individual mice over time. Three tumors from the anti–PD-1 group failed to grow tumors and are overlapped on the *x* axis of panel **B**. We analyzed differences in tumor growth data by logistic regression. Survival curves demonstrate that anti–PD-1 treatment significantly prolonged survival for mice with E0771 tumors (**C**) but not with AT-3 (**F**), as analyzed by the Mantel-Cox test.

To study cholesterol uptake and the immunotherapy response in tumor-infiltrating T cells, we utilized cell lines that model TNBC but exhibit different responses to immunotherapy. EO771 cells, one of the only mammary tumor cell lines derived from spontaneous tumorigenesis in C57BL/6J mice, responds to immunotherapy and has been used to test effects of therapeutic agents to improve immunogenicity (14). In contrast, AT-3 cells are derived from transgenic MMTV-PyMT mice and respond poorly to immunotherapies, including anti–PD-1, despite the presence of intratumoral T cells (15, 16). To directly compare effects of anti–PD-1 antibody therapy on tumor growth, we orthotopically implanted either EO771 or AT-3 breast cancer cells with syngeneic immortalized mouse mammary fibroblasts. Three days after implanting cells, we randomly assigned mice to treatment with anti–PD-1 antibody therapy or vehicle. Mice received a total of 4 doses administered every 3 days. We quantified tumor size by caliper measurements and monitored survival until mice reached humane endpoints for tumor burden. Mice with EO771 tumors showed remarkable reductions in tumor growth (*P* = 0.0001) and significantly increased overall survival in response to anti–PD-1 therapy (*P* = 0.031) (Figure 1, A–C). By comparison, anti–PD-1 treatment elicited no change in tumor growth (Figure 1, D and E) or survival over control in mice with AT-3 tumors (Figure 1F). These data validate responsive and treatment refractory mouse models of immunotherapy in TNBC, providing contrasting systems to investigate cholesterol as a marker of T cell activation.

**Figure 2:**
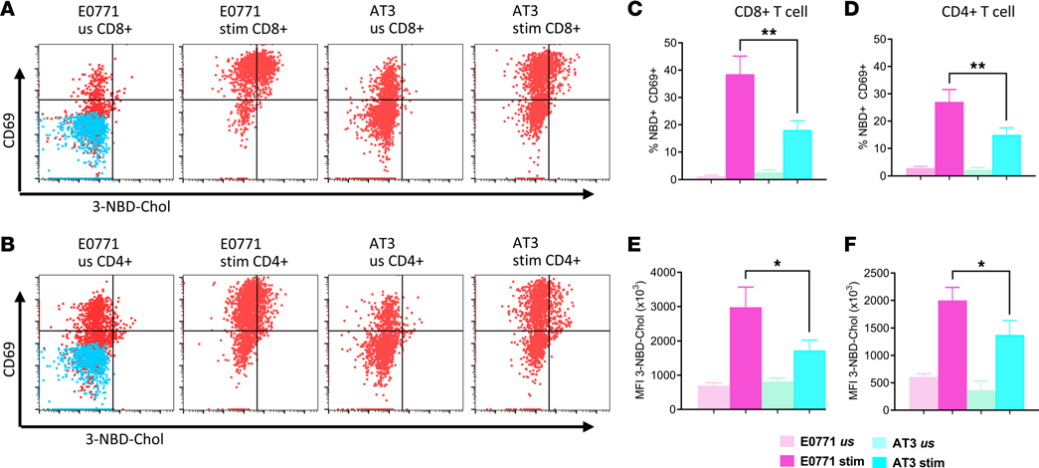
T cells from ICI-responsive versus -nonresponsive tumors have greater uptake of fluorescent cholesterol. T cells were isolated from ICI-responsive EO771 or ICI-nonresponsive AT-3 tumors and left unstimulated (us) or restimulated on anti-CD3e–coated (stim) dishes for 18 hours in the presence of 3-NBD–labeled cholesterol. We determined uptake of labeled cholesterol in activated, CD69^+^ CD8^+^ (**A**) or CD4^+^ (**B**) T cells by flow cytometry. CD8^+^ (**C**) and CD4^+^ (**D**) T cells from EO771 tumors show significantly greater percentages of CD69^+^ cells with uptake of 3-NBD cholesterol than from ICI-nonresponsive AT-3 tumors. Stimulated CD8^+^ (**E**) and CD4^+^ (**F**) T cells from EO771 tumors also exhibited significantly higher mean fluorescence intensity (MFI) for cholesterol uptake. Data are combined from 2 experiments. *P < 0.05, **P < 0.01 by 2-tailed Student’s *t* test (**C** and **D**) or nonparametric Mann-Whitney test (**E** and **F**).

We measured uptake of cholesterol in T cells isolated from EO771 or AT-3 breast tumors and restimulated ex vivo in the presence of fluorescent 3-NBD-cholesterol. Due to the location and orientation of the NBD tag, this fluorescent cholesterol models cholesterol orientation in membranes better than previous fluorescently tagged cholesterol analogs (17). Solid tumors rarely contain naive T cells, so ex vivo tumor-infiltrating T cells were stimulated with low-level CD3ε for 18 hours (18). We assessed uptake of 3-NBD-cholesterol in combination with the activation marker CD69 on T cells using spectral cytometry (Figure 2, A and D). Cholesterol uptake correlated significantly with expression of the activation marker, CD69 (*P* = 0.0151) (Figure 2, B and C). Additionally, cholesterol uptake increased to a markedly greater extent in CD8^+^ (*P* = 0.026) and CD4^+^ (*P* = 0.0286) T cells from EO771 tumors when compared with T cells from AT-3 (nonresponsive) tumors (Figure 2, E and F).

**Figure 3:**
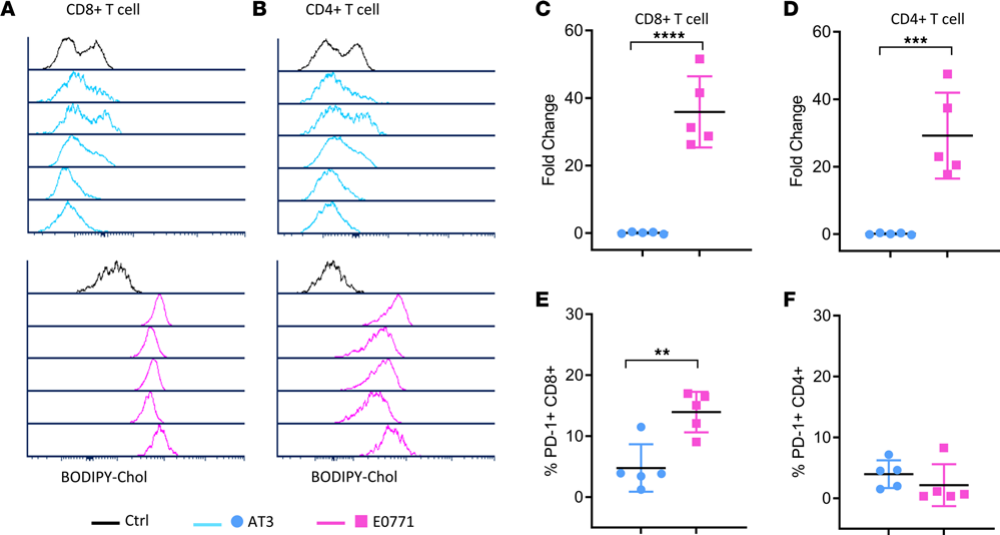
T cells in ICI-responsive EO771 tumors show greater uptake of fluorescent cholesterol in vivo. We injected C57BL/6J mice intraperitoneally with cholesterol labeled with BODIPY and euthanized animals 24 hours later to collect and dissociate tumors for flow cytometry (*n* = 5 each for EO771 and AT-3). Plots for (**A**) CD8^+^ and (**B**) CD4^+^ T cells show accumulation of BODIPY-cholesterol in cells from individual tumors from EO771 and AT-3 tumors relative to vehicle only or isotype antibody control. (**C**) CD8^+^ and (**D**) CD4^+^ T cells in EO771 tumors showed significantly higher fold change accumulation of fluorescent cholesterol relative to FMO control. (**E**) CD8^+^, but not (**F**) CD4^+^, T cells in EO771 tumors also expressed higher levels of PD-1. **P < 0.01,***P < 0.001, ****P < 0.0001 for differences between means using nonparametric Mann-Whitney tests (**C** and **D**; *n* = 5 mice per group), while differences between T cell population percentages were assessed using 2-tailed Student’s *t* test (**E** and **F**).

To assess uptake of cholesterol in T cells in vivo, we injected tumor-bearing mice with a fluorescent BODIPY–labeled cholesterol. BODIPY-labeled cholesterol effectively partitions with intracellular cholesterol, remains stable in vivo, and exhibits greater fluorescence than the 3-NBD label (19). Twenty-four hours after injecting BODIPY-cholesterol, we collected intratumoral T cells for flow cytometry. T cells from responsive EO771 tumors showed a higher uptake of labeled cholesterol when compared with AT-3 tumors, as seen in histograms (Figure 3, A and B) and normalized BODIPY mean fluorescence intensity (MFI) in both CD8^+^ (*P* < 0.0001) and CD4^+^ (*P* = 0.0009) T cells (Figure 3, C and D). Prior studies reported increased expression of PD-1 on T cells that take up more cholesterol. Indeed, PD-1 expression on T cells increased significantly on CD8^+^ T cells from EO771 tumors with higher cholesterol uptake (*P* = 0.004) (Figure 3E). Modulation of PD-1 did not occur on CD4^+^ T cells (Figure 3F). Together, these experiments demonstrate increased uptake of cholesterol in T cells from tumors that respond to immunotherapy.

### Activated T cells increase cholesterol uptake.

Cholesterol is required for cell division processes, and its presence in the plasma membrane of T cells stabilizes TCR nanoclusters to enhance overall activation (20). Cholesterol uptake as related to cell status in T cells remains a controversial topic. One study found that hypercholesteremia in T cells reflected proliferation of T cells (21). In contrast, elevated cholesterol in T cells also reportedly reflects exhaustion (7). To further investigate to what extent levels of cholesterol change with activation, we treated naive mouse T cells with increasing amounts of anti-CD3ε and proportional increases in anti-CD28 antibodies (3 times the anti-CD3ε concentration). Using an in vitro assay to quantify total cholesterol, free cholesterol, and cholesterol esters, we established that activation increased total cholesterol concentration in T cells, with differences between 0 μg/mL and 1 μg/mL or 10 μg/mL of anti-CD3e (Supplemental Figure 1 and Supplemental Figure 2A; supplemental material available online with this article; https://doi.org/10.1172/jci.insight.175320DS1). The concentration of cholesterol esters did not change with increasing activation in T cells after 24 hours (Supplemental Figure 2A). While this assay demonstrates that total cholesterol increased in T cells after TCR activation, these data do not distinguish between synthesis and uptake of cholesterol.

To selectively measure uptake of cholesterol during T cell activation, we leveraged a PET radiotracer analog of cholesterol, eFNP-59, developed by our research group. Our group has validated eFNP-59 as an imaging probe for cholesterol trafficking and uptake in humans (10). Because we added eFNP-59 to the culture medium, any accumulated radiotracer in T cells represents uptake, rather than de novo synthesis, during activation. We incubated activated T cells with eFNP-59 according to the diagram in Supplemental Figure 2B. After washing cells to remove extracellular radiotracer, we used autoradiography with a phosphor imaging screen to measure uptake by T cells. Phosphor imaging allows scalability of samples (directly in 96-well plates) while maintaining sensitivity with a large linear range of detection. After 1 hour of incubation, uptake of eFNP-59 increased progressively with higher concentrations of activating antibodies. Each T cell treatment displayed significant differences between levels of anti-CD3 stimulation with unique EC_50_ values when tested against all data points in logistical nonlinear regression (*P* < 0.0001) (Supplemental Figure 2C and Supplemental Figure 3).

To extend these findings to humans, we treated activated human T cells from peripheral blood with eFNP-59, as outlined in Supplemental Figure 2B. We found that human T cells also take up eFNP-59 when activated, but they were less sensitive to exogenous cholesterol uptake when compared with mice (*P* < 0.0001) (Supplemental Figure 2D). To study further the impact of cholesterol trafficking on the human immune system, we analyzed data from the Immune Cell Atlas, originating from Martin et al. and hosted by the Broad Institute (22). These data comprise an annotated set of immune cells from an uninflamed lamina propria, an excellent tissue site for studying varied immune responses without the presence of disease. From this data set, we utilized the T cell clusters previously annotated by gene expression (Supplemental Figure 2E). Genes responsible for uptake and trafficking of cholesterol displayed increased relative expression in the cycling cluster compared with other clusters (Supplemental Figure 2F). These included genes specifically annotated for cholesterol uptake and trafficking, including scavenger receptors like *SCP2* and *SCARB1*, endosome-associated proteins such as *STARD3NL*, *NPC1*, *NPC2*, and transcription factor *SREBF2* (23–27). Additional upregulated genes, *COMMD1*, *WASH1*, *ANXA2*, and *LAMTOR1*, function in cholesterol uptake and trafficking as well as other cellular processes (28–31). Increased expression of genes related to cholesterol uptake and trafficking in activated human T cells supports translatability of these findings.

### Uptake of eFNP-59 in T cells is elevated with activation and immunotherapy in mouse TNBC.

**Figure 4:**
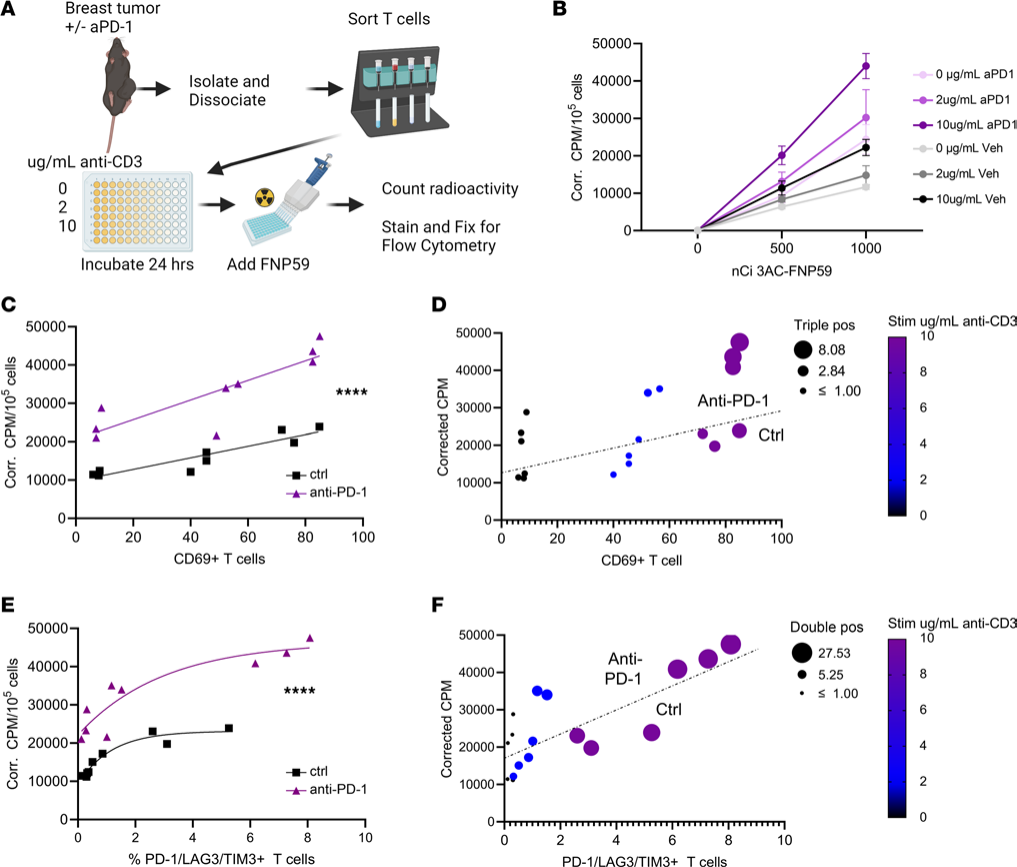
eFNP-59 uptake in T cells correlates directly with activation, differing from exhaustion. (**A**) Mice were inoculated with E0771 and treated with and without anti–PD-1 immunotherapy. T cells were extracted, activated, and activation status compared to cholesterol uptake as in the diagram. (**B**) Cholesterol uptake was first compared between activation and immunotherapy groups with different amounts of eFNP-59. Using the data from the 1000-nCi treatment, (**C**) T cell activation marker CD69 was compared to normalized activity in a scatter plot and (**D**) multiparametric plot also assessing activation signal with pseudocolor scale, estimation of triple-positive T cells with bubble scale, and information on immunotherapy status (samples above or below dashed line). (**E**) Normalized activity (cholesterol uptake) was directly compared to triple-positive T cells and then again as (**F**) a multiparametric plot with pseudocolor plot for double-positive T cells, bubble size for anti-CD3 stimulus, and immunotherapy status (above or below dashed line). ****P < 0.0001 by least squares fit.

To assess the relationship of eFNP-59 uptake in activated versus exhausted T cells, we injected mice with EO771 cells and treated animals with anti–PD-1 or vehicle, following the protocol in Figure 1. We euthanized mice 4 days after the final injection. Following isolation of TILs from the tumor, we reactivated these cells with a moderate stimulus (2 μg/mL anti-CD3), a strong exhausting stimulus (10 μg/mL anti-CD3), or control stimulus for 1 day. We then incubated cells with eFNP-59, quantified radioactivity normalized to numbers of cells, and stained/fixed cells for downstream analyses (Figure 4A). Radioactivity in each group increased based on dose of anti-CD3 with cells from mice treated with anti–PD-1, exhibiting greater uptake than mice treated with vehicle control for each stimulus (Figure 4B). When comparing each treatment group versus amount of added eFNP-59, the 1000-nCi dose showed best separation for further analyses (Figure 4B). We identified a linear relationship between uptake of eFNP-59 in T cells for the 1000-nCi dose versus levels of the activation marker CD69, with significant separation between treatment groups (*P* < 0.0001) (Figure 4C). A multiparametric display also demonstrated that exhaustion markers TIM3 and LAG3 also increased as radioactivity and activation signal increased (Figure 4D). To better compare uptake of eFNP-59 in activated versus exhausted T cells, we plotted triple-positive exhausted T cells (PD-1^+^LAG3^+^TIM3^+^) against radioactivity (Figure 4E). This relationship showed a more logarithmic plateau in which moderate and strong activation signals produced similar measures of exhausted triple-positive T cells when compared with uptake of eFNP-59 (Figure 4E). We also compared double-positive T cells (cells with any combination of 2 positive markers for PD-1, LAG3, or TIM3 that are less exhausted but activated) with uptake of eFNP-59. Populations of double-positive cells and uptake of eFNP-59 increased to a greater extent with increasing stimulus (Figure 4F). Overall, we found that cholesterol uptake maintained a more linear relationship to T cell activation than exhaustion, and anti–PD-1 immunotherapy improved overall uptake of eFNP-59 in functioning activated T cells.

**Figure 5:**
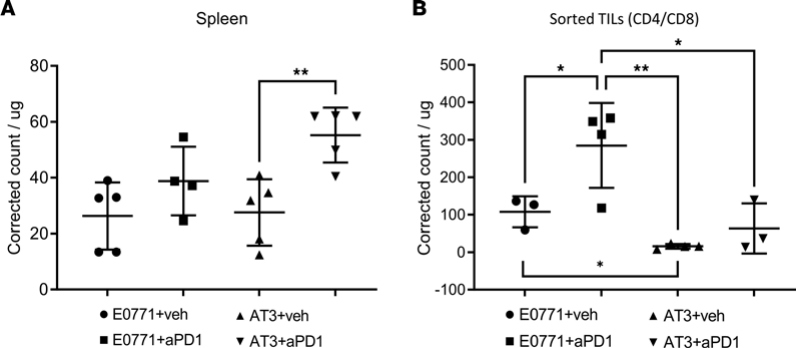
T cells in ICI-responsive tumors had greater uptake of eFNP-59. When tumors reached approximately 70 mm^2^, we treated mice with anti–PD-1 antibody or control for 4 days. Mice were then injected with 100 μCi of eFNP-59 followed by T cell isolation protocols. (**A**) Graph shows uptake of eFNP-59 per microgram of spleen tissue measured by scintigraphy. Symbols show individual mice with annotations for mean values and standard deviations. (**B**) We isolated CD4^+^ and CD8^+^ tumor-infiltrating lymphocytes (TILs) by positive selection with immunomagnetic beads and determined accumulation of eFNP-59 normalized to total cell protein with a BCA assay. Representative data from 2 experimental replicates, with statistical comparisons by 1-way ANOVA with Dunn’s multiple-comparison test. *P < 0.05; **P < 0.01.

To test uptake of eFNP-59 in T cells in vivo and use of this radiotracer to predict and monitor response to immunotherapy, we established mice with EO771 or AT-3 breast tumors. After tumors reached approximately 7 mm in diameter, we randomly assigned mice to treatment with anti–PD-1 or vehicle. We hypothesized that anti–PD-1 treatment would further alter cholesterol uptake, with reinvigoration of endogenous T cells after a single dose of ICI. Four days after treatment, we intravenously injected mice with 100 μCi of eFNP-59. We chose to analyze cholesterol uptake 4 days after anti–PD-1 treatment to allow the ICI treatment to impact T cell activation/function before measuring cholesterol uptake. We quantified uptake of eFNP-59 in total dissociated splenocytes, and in T cells recovered from dissociated tumors. Splenocytes from mice treated with vehicle only accumulated comparable amounts of eFNP-59 for both the EO771 and AT-3 groups (*P* = 0.0039) (Figure 5A). Relative to vehicle only, anti–PD-1 treatment modestly increased radiotracer uptake in splenocytes from both groups, although amounts differed significantly only for mice with AT-3 tumors (*P* = 0.0039). Isolated T cells from the vehicle-treated EO771 tumors accumulated more eFNP-59 than vehicle-treated AT-3 tumors, reproducing results with fluorescent cholesterol analogs (Figure 5B). Furthermore, T cells recovered from anti–PD-1–treated EO771 tumors had significantly greater uptake of eFNP-59 than anti–PD-1–treated AT-3 tumors (*P* = 0.01) (Figure 5B). These experiments demonstrate that (a) baseline uptake of eFNP-59 before ICI treatment correlates with response to therapy in these models, and (b) change in uptake of this radioactive cholesterol analog reflects T cell response to ICI.

### Cycling T cells in human TNBC tumors maintain elevated expression of cholesterol trafficking genes.

**Figure 6:**
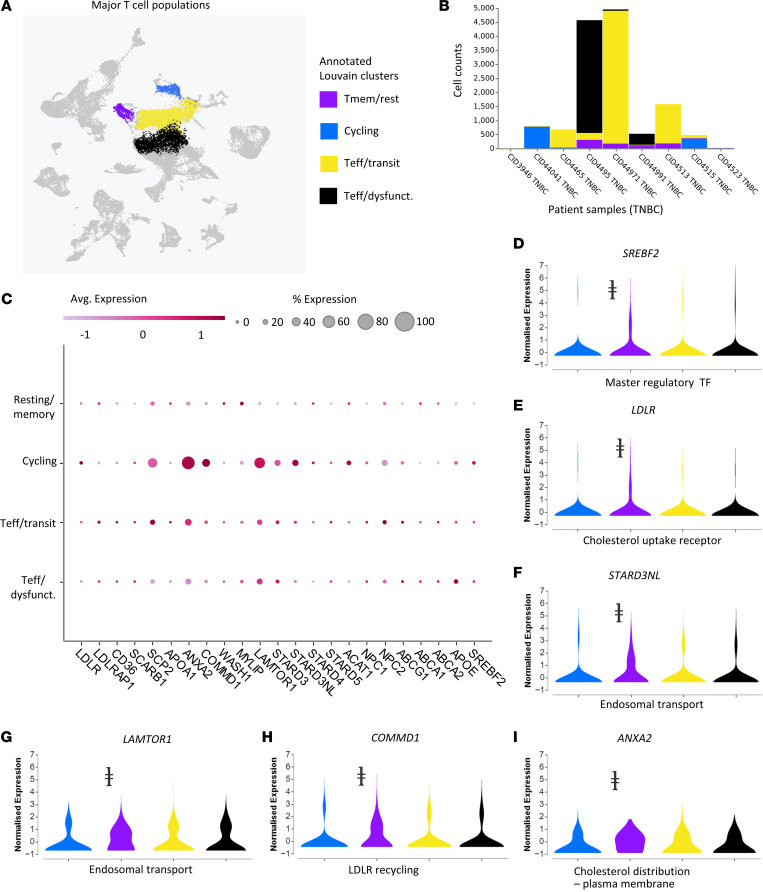
Cycling T cell populations in patients with TNBC upregulate genes related to cholesterol metabolism. (**A**) Reanalysis of single-cell RNA sequencing data (29) with Cellenics software displays Louvain clusters of annotated T cell states, including custom cell sets derived from these clusters: T memory/resting, highly activated, T effector/transitional state, and T effector/dysfunctional. (**B**) Plot shows proportions of the absolute counts across for various T cell subsets across TNBC patients (*n* = 8429 cells from 9 tumors). (**C**) Clustered averaged gene expression data reveal upregulation of relevant genes involved in uptake and intracellular trafficking of cholesterol in cycling T cells. (**D**–**I**) Violin plots of normalized expression of specific genes: (**D**) *ANXA2* (cholesterol distribution to the plasma membrane), (**E**) *LAMTOR1* (endosomal transport), (**F**) *STARD3NL* (endosomal transport), (**G**) *COMMD1* (LDLR recycling), (**H**) *LDLR* (cholesterol uptake), and (**I**) *SREBF2* (positive regulation of cholesterol uptake and synthesis). The ⱡ symbol indicates *P* < 10^–20^ between the cycling cluster and other T cell clusters, as determined by Welch’s *t* test.

To translate these discoveries to human breast cancer, we queried publicly available data sets and analyzed single-cell RNA sequencing (scRNA-seq) data from human TNBC (32). Louvain clusters of TNBC patient cells without additional characterization or bias reported 4 major T cell states (Figure 6A). These states included resting/naive T cells, highly activated/cycling T cells, transitional effectors (toward exhaustion), and exhausted effectors. We annotated T cell states based on highly expressed genes in each Louvain cluster: (a) cycling T cells are defined by the high expression of genes related to microtubule reorganization and mitosis, including upregulation of *STNM1* and *MKI67*; (b) transitional state cytotoxic T cells based on high expression of *GZMK* and moderate levels of *PDCD1* and *HAVRC2*; (c) dysfunctional T cells classified by high expression of *PDCD1*, *HAVCR2*, and *CXCL13* plus expression of *CSF1*; and (d) resting memory T cells defined by high expression of *CXCR4* and ribosomal proteins (33–35). Cell counts of clusters from each patient revealed that patients with large T effector populations (whether transitional or exhausted) also carried a smaller population of actively proliferating T cells (Figure 6B). We determined and annotated T cell states of each cluster using top gene expression of the Louvain clusters (Figure 4B). Cycling T cells displayed the greatest expression of genes directly and indirectly involved in cholesterol uptake and trafficking (Figure 6C). Furthermore, genes involved in cholesterol distribution (Figure 6D), endosomal transport (Figure 6, E and F), receptor recycling (Figure 6G), receptor expression (Figure 6H), and transcription factor control (Figure 6I) were modulated in the cycling T cell populations when compared with other clusters (Supplemental Table 1 for all comparisons). When analyzed using Enrichr, we found upregulation families of genes related to “Regulation of Cholesterol Biosynthesis By SREBF” and “Activation Of Gene Expression By SREBF” in the cycling T cell cluster (Supplemental Table 2). Overall, these data show some patients with TNBC develop tumors that produce a population of cycling T cells (often accompanied by an expanded population of effector T cells; Figure 6B), with features of gene expression indicative of cholesterol uptake and trafficking. Together, these data suggest that increased uptake of cholesterol demarks activated, antitumor T cells in human TNBC.

## Discussion

Tumors can generally present as (a) an immune-desert phenotype lacking immune cells and immune signaling; (b) an immune-excluded phenotype in which immune cells encounter mechanical and cell signaling barriers that prevent tumor infiltration; or (c) immune-infiltrated or inflamed phenotypes (36). TNBC in humans is heterogeneous, but presents with an inflamed microenvironment more frequently than other subtypes of breast cancer. Although generally considered more inflamed, TNBC can exist anywhere on this continuum of immune phenotypes and exhibits substantial intra- and intertumoral heterogeneity. Tumors with high infiltration of lymphocytes such as NK cells, cytotoxic T cells, and B cells correlates with more favorable prognosis (37). A key publication from Hu et al. that has guided immunotherapy clinical trials and practice in TNBC identified 3 major immune subtypes (38). Only the subtype with enrichment of lymphocytes and immune checkpoint ligands showed favorable outcomes (38). However, even the most favorable subtype of TNBC demonstrated heterogeneity in TIL function and overall efficacy (through modulation of key genes). Clinical trials and treatment with immunotherapy rely on a combined positive score (CPS) to subclassify patients and predict response (3). The CPS system analyzes numbers of PD-L1–positive (or other marker) tumor cells, lymphocytes, and macrophages out of the total number of viable cancer cells in histologic sections from biopsies. While used widely, the CPS may vary based on selection of different antibodies for immunostaining, and CPS cutoffs have only modest success in predicting response to ICIs (39). Therefore, identification of more informative and potentially noninvasive biomarkers for efficacy of ICIs remains a highly active area of translational research (40).

Given past evidence linking T cell activation with increased cholesterol, we investigated uptake of cholesterol as a marker for efficacy of immunotherapy in mouse models of TNBC. We discovered that increasing levels of TCR activation correlated with higher uptake of cholesterol in T cells. Capitalizing on this observation, we leveraged eFNP-59, a validated radiotracer for imaging cholesterol trafficking and uptake in humans, as a marker for efficacy of ICI. For tumor studies, we utilized 2 mouse models of TNBC, EO771 and AT-3, that are responsive or not responsive to immunotherapy, respectively. Ex vivo–restimulated and in vivo T cells from ICI-responsive breast tumors in mice accumulated substantially more cholesterol than T cells from ICI-nonresponsive tumors. Remarkably, we detected uptake of eFNP-59 within 3 hours of injection in tumor-bearing mice. T cells from tumors responsive to ICI therapy showed higher uptake of eFNP-59 under basal conditions and following anti–PD-1 therapy. In human patients with TNBC, populations of activated T cells exhibited gene signatures of enhanced cholesterol uptake as compared with resting, effector, and exhausted T cell states. This gene signature included upregulation of key genes, including *ANXA2*, a multifaceted protein that works with *CD36* for lipid scavenging (41). Additionally, we found upregulation of the master transcriptional regulator of cholesterol metabolism, *SREBF2* (42).

Determining which patients will benefit from immunotherapy remains a central obstacle to effective use of these drugs for cancer. There are currently 3 FDA-approved predictive markers for immunotherapy: PD-L1 expression, tumor mutational burden (TMB), and microsatellite instability (MSI). Approved methods rely on tumor biopsies, limiting the ability to repeatedly monitor tumor evolution and response to therapy over time. Several other investigational approaches exist, including gene profiles for T cells and presence of B cells (43). Listed strategies focus on predicting response to immunotherapy to guide selection of patients. However, none of these methods offer the important capability to detect response to therapy early in treatment.

Toward the goal of assessing response to ICIs, researchers have developed PET imaging probes based on antibodies, antibody fragments such as affibodies, or small molecules for the PD-L1/PD-1 pathway or other ICIs (44, 45). PET imaging studies in mouse models or early clinical studies reveal heterogeneous expression of immune checkpoint proteins in primary tumors and various metastases, emphasizing the value of imaging to detect intrapatient heterogeneity that could affect treatment outcomes (46). As discussed previously, detecting expression of PD-L1 protein or other immune checkpoint molecules at least by immunostaining has shown limited efficacy in predicting patients who will respond to ICIs. Imaging probes for cell-surface ICIs identify levels of specific targets, and imaging after starting therapy can detect to what extent an ICI blocks the target molecule. A key limitation is that these probes do not detect to what extent a therapy activates functional T cells, the key effector population of current ICIs. Imaging with eFNP-59 offers a potential noninvasive PET approach to assess response to checkpoint inhibitor immunotherapy. We note our current study did not longitudinally measure changes in uptake of eFNP-59 during ongoing ICI therapy. Future, larger scale studies will investigate eFNP-59 as a potential imaging biomarker to evaluate efficacy of therapy and help optimize treatment protocols. While our study provides valuable insights about cholesterol and T cell activation during immunotherapy, we acknowledge potential variations between human and mouse cells regarding the sensitivity of eFNP-59 to ICI responses. However, given our group’s prior work with eFNP-59 in humans (10), translation to human study is readily possible and being planned.

Functions of cholesterol in T cells and antitumor immunity remain controversial. One study found that hyperlipidemia and increased cholesterol plasma disrupted T cell homeostasis and drove expansion of effector memory T cells (21). In contrast, Xingzhe Ma et al. found that cholesterol in the tumor microenvironment correlated with T cell exhaustion due to ER stress (7). An alternative or additional hypothesis is that the presence of exhausted T cells indicates a prior or active immune response in those tumors. Notably, the study by Ma et al. did not show to what extent the effect of cholesterol on T cells reversed with immunotherapy. In our study, we found that cholesterol uptake had a stronger linear association with markers for T cell activation than T cell exhaustion, further strengthening our hypothesis that cholesterol uptake is more of a surrogate for active T cell responses rather than terminal states of antitumor immunity. Given the noninvasive nature of imaging, studies with eFNP-59 can be performed over time during therapy to help investigate functions of cholesterol in tumor immunity and explain the results of prior work where questionable contradictions exist. A recent study comprehensively mapped cholesterol metabolism and disposition in tumor environments (47). Intratumoral T cells showed cholesterol deficiency, which impaired proliferation and triggered autophagy-mediated cell death. Across all cells in a tumor, tumor cells and myeloid lineage cells contained the highest amounts of cholesterol, with lower overall levels in T cells. Intriguingly, suppressing cholesterol efflux pathways by targeting the transcription factor LXRβ enhanced antitumor functions of T cells. These data support our findings that activated T cells prefer to accumulate more cholesterol and point to the need for methods to track disposition of cholesterol in tumor environments. Methods used in this study focused on uptake of cholesterol in relation to effects of immunotherapy. Our data do not address to what extent, if any, overall increases in cholesterol or cholesterol metabolism in T cells directly regulate antitumor immunity.

eFNP-59 has limitations for PET imaging of ICIs in cancer because uptake of the radiotracer and cholesterol in a tumor is not restricted to T cells. Signal from eFNP-59 would also include uptake of cholesterol by macrophages, cancer cells, and other cell types. Although this nuance adds a layer of complexity to the potential translation of our findings to in vivo scenarios, similar challenges are present with interpreting FDG during immunotherapy. Notably, monitoring changes over time within the tumor or monitoring T cells in blood could provide valuable insights in future studies. Potentially, measuring an increase in eFNP-59 uptake in a tumor (normalized to tumor volume) between imaging studies conducted before and shortly after starting ICI treatment could reflect accumulation of reactivated T cells. Other immune cells potentially also could contribute to an increase in uptake in the short term, although such changes still would be consistent with a more inflamed tumor environment. From our study, we can conclude that ex vivo analysis of this PET radiotracer could be used to measure redistribution of cholesterol in a tumor in preclinical models and/or biopsy samples.

Additionally, while our primary emphasis is on PET tracers like eFNP-59, we utilized available optical imaging agents (fluorescent cholesterol) due to logistical constraints on availability of the radiotracer and as an independent measure of cholesterol utilization. Although fluorescence-based imaging has a lower sensitivity compared with PET tracers, it can provide relative quantification of differences in uptake.

eFNP-59 presents numerous benefits over the previous iodinated version of the compound. The poor safety profile, onerous synthetic protocols, and poor image quality restricted the iodinated version (^131^I-NP59) of this tracer to detecting diseases of the adrenal cortex. Use of ^131^I-NP59 has been discontinued in the United States (42). eFNP-59 overcomes many of these limitations of the previous generations of this tracer, and initial studies in humans show ideal imaging characteristics and no toxicity. Although other tools (such as mass cytometry) may also provide insights into T cell activation through surrogate cell markers, metabolic changes such as uptake of cholesterol in T cells offer a more direct indication of their activation status and functionality. Our radiotracer, eFNP-59, not only benefits from an established safety profile in humans but also exhibits heightened sensitivity for detection. In situations where subtle changes necessitate enhanced sensitivity, our radiotracer may offer a valuable alternative. These studies provide a roadmap for future studies and potential clinical translation of this radiotracer for monitoring treatment with ICIs in a wide variety of cancers.

Overall, efficacy of eFNP-59 for identifying activated, antitumor immune cells in mice, combined with relevance of cholesterol uptake in activated T cells in humans, support future testing of this radiotracer to stratify patients and monitor response to ICIs in TNBC and possibly other malignancies. This radiotracer could be used in TNBC before biopsy or surgery, followed by isolation of T cells and ex vivo imaging of accumulation. Alternatively, since early responses to ICI therapy include activation of peripheral blood CD8^+^ T cells enriched for antitumor T cell clones, changes in accumulation of radiotracer in blood samples collected before and after treatment could monitor efficacy of these drugs. Future studies will investigate these potential applications of eFNP-59 for prediction and treatment monitoring with ICIs in mouse models of breast cancer and ultimately patients.

## Methods

### Sex as a biological variable.

In this study, we utilized female mice due to the commonality of breast cancer in female humans. It is unknown whether the findings are relevant for male mice.

### Cell lines.

We cultured mouse EO771 (ATCC) and AT-3 (EMD Millipore) breast cancer cells and C57BL/6J mouse mammary fibroblasts (gift of Harold Moses, Vanderbilt University) in DMEM base media (Gibco, 11965) supplemented with 10% FBS, 1% GlutaMax (Thermo Fisher Scientific, 35050061), and 1% penicillin-streptomycin (Gibco, 15140148). We maintained all cells in a humidified 37°C incubator with 5% CO_2_.

### Mouse tumor models.

We implanted 5 × 10^5^ EO771 or AT-3 mouse mammary tumor cell lines and 1 × 10^5^ mouse mammary fibroblasts orthotopically into fourth inguinal mammary fat pads of 6- to 8-week-old female C57BL/6J female mice (The Jackson Laboratory) (48). We housed mice in specific pathogen–free housing facilities maintained by the Unit for Laboratory Animal Medicine at the University of Michigan. Three days after implanting cells, we randomly assigned mice to treatment with anti–PD-1 antibody (10 mg/kg intraperitoneal) (BioXCell, CD279) or isotype/vehicle control administered every 3 days for 4 total doses. We measured growth of orthotopic tumors with calipers twice per week, quantifying tumor size for each mouse as mm^2^ by multiplying orthogonal length × width measurements. We euthanized mice at a predetermined humane endpoint of tumor size, which defined time of censoring for survival analyses.

### T cell assay preparation, reagents, and antibodies.

We purified T cells from the spleens of wild-type C57BL/6J mice by processing tissue through a 70-μm filter, lysing red blood cells with ACK buffer (Thermo Fisher Scientific, A1049201), and sorting cells using the Pan T Cell Isolation Kit II (Miltenyi Biotec, 130-095-130). For ex vivo activation of T cells, we coated 96-well plates with indicated amounts of anti-CD3ε antibody supplemented with soluble anti-CD28 at 2 times the amount of anti-CD3ε and 5 U/mL IL-2 (BioLegend). For activation, specific antibodies included anti–mouse CD3ε (clone 145-2C11) and CD28 (clone 37.51) or anti–human CD3 antibody (clone OKT3) and anti–human CD28 (clone CD28.2) (antibodies from BioLegend). For flow cytometry experiments, antibodies included anti–mouse CD45 (clone S18009D), TCRβ chain (clone H57-597), CD3 (clone 17A2), CD8α (clone 53-6.7), CD4 (clone RM4-5), CD69 (clone H1.2F3), and CD279 (clone 29F.1A12) (BioLegend). In follow-up ex vivo T cell experiments with eFNP-59, we added antibodies for TIM3 (clone RMT3-23) and LAG3 (clone C9B7W) to further compare exhaustion. Cholesterol probes included cholesteryl BODIPY 542/563 C11 (Invitrogen, C12680) and 3-dodecanoyl-NBD cholesterol (Cayman Chemicals, 13220) for in vivo and in vitro experiments, respectively.

### Synthesis of eFNP-59.

We synthesized eFNP-59 as previously described (10). Briefly, ^18^fluoride was produced via the ^18^O(p,n)^18^F nuclear reaction with a GE PETtrace cyclotron equipped with a high-yield fluorine-18 target. ^18^Fluoride was delivered in a bolus of [^18^O]H_2_O to the synthesis module and trapped on a QMA-Light sep-pak cartridge to remove [^18^O]H_2_O. ^18^Fluoride was then eluted into the reaction vessel with potassium carbonate. Acetonitrile with Kyrptofix 2.2.2 (Sigma-Aldrich, 8106478250) was added to the reaction vessel, and the ^18^fluoride was azeotropically dried by heating the reaction vessel to 100°C and drawing full vacuum. After this time, the reaction vessel was subjected to both an argon stream and a simultaneous vacuum draw at 100°C. The solution of precursor in DMSO was added to the dried ^18^fluoride, and it was heated at 120°C with stirring for 20 minutes. The ester group was removed by treatment with KOH and heating at 110°C for 25 minutes. eFNP-59 was isolated via reverse-phase chromatography and reformulated into a solution of saline, ethanol, and Tween 80.

### In vitro/in vivo uptake of eFNP-59.

For experiments measuring T cell accumulation of eFNP-59 in vitro, we washed 96-well plates with activated cells once before adding radiotracer at indicated concentrations (*n* = 3 wells per concentration). We incubated plates for 1 hour and then washed twice with RPMI medium (Gibco, 11875903) containing 0.1% serum before quantifying retained radioactivity in each well. We performed radioactivity measurements for experimental wells and a standard curve with a phosphor imaging screen (GE Typhoon FLA 7000) or gamma counter (2470 Automatic gamma counter- Wizard2, Perkin Elmer). Image analysis for phosphor imaging was performed using ImageQuant (Molecular Dynamics) software. Regions of interest were drawn and converted to disintegrations per minute (DPM) and specific activity using molecular standards on the same plate and data were further modeled and plotted with GraphPad Prism v9. Gamma counts were corrected using Radprocalculator software (http://www.radprocalculator.com/) and normalized to cell counts or mass as indicated. For exhaustion/activation studies, T cells were stained for flow cytometry (see above) and fixed after obtaining radioactive counts.

For mouse experiments, we intravenously injected 100 μCi of eFNP-59 3 hours before euthanizing mice to recover spleen and tumor-infiltrating T cells. To recover T cells for analysis, we dissociated tumors in 5 mL of tumor dissociation buffer containing RPMI base media, 5% FBS, 300 U/mL collagenase I (Thermo Fisher Scientific), 150 U/mL collagenase IV (Thermo Fisher Scientific), and 200 μg/mL DNase I (Roche), using the TDK1 program on the gentleMACS Octo Dissociator (Miltenyi Biotec). After 30 minutes of incubation, we passed tumor tissue through a 70-μm filter and isolated T cells using CD4/CD8 (TIL) MicroBeads and cell sorting magnets from Miltenyi Biotec. Recovered T cells were transferred into 1.5 mL tubes and decay-corrected radioactive counts with an automatic gamma counter were obtained. We quantified accumulation of eFNP-59 in excised whole spleens and normalized accumulated radiotracer activity to total protein via BCA assay (Pierce) for T cells and to whole organ weight for the spleen.

### In vitro/in vivo fluorescent cholesterol uptake and flow/spectral cytometry.

For in vitro experiments measuring uptake of fluorescent cholesterol analogs during early T cell activation, we added 1 μg/mL 3-NBD-cholesterol to 96-well plates with T cell activation conditions for 18 hours. We washed T cells once with PBS prior to staining in the plate using antibodies against T cell identification and activation markers described above. We analyzed cells on the same day without fixing using a Bio-Rad Ze5 Analyzer. Single-color controls enabled compensation and spectral unmixing of samples, and fluorescence minus one (FMO) control ensured no autofluorescence or incidental FRET with emission from BODIPY. We used the following gating strategy: (a) FSC/SSC to exclude debris; (b) single cells defined by FSC-H/FSC-A gated on the linear singlet population; (c) live cell populations using live-dead exclusion dye (Zombie Aqua, 423101, Bio-Rad); (d) CD45^+^ to include only white blood cells; (e) TCRβ constant chain–positive to include all T cells; and (f) CD4 or CD8 gates and finally analytic antibodies or probes, including CD69, PD-1, or cholesterol.

To analyze total T cell cholesterol content, we used the Cholesterol/Cholesterol Ester-Glo Assay from Promega. We set up experiments in 96-well plates, with 1 × 10^5^ cells per well. Cells were stimulated with plate-bound anti-CD3 using 0.01, 0.1, 1, and 10 μg/mL. An additional group received no stimulation. We treated all groups with soluble anti-CD28 at 3 times the amount of anti-CD3. We excluded IL-2 to prevent the impact of rapid growth stimulation and dissociate the effect from added growth factor/high-affinity IL-2 receptor expression. After 24 hours, we diluted samples in Cholesterol Lysis Solution and followed the manufacturer’s procedure with 1:8 and 1:20 dilutions of sample. This protocol measures total cholesterol with samples assayed plus esterase in the Cholesterol Detection Reagent and free cholesterol with samples assayed minus esterase in the Cholesterol Detection Reagent. Cholesterol ester levels are calculated as the difference between total and free cholesterol. We measured luminescence on an IVIS Lumina LT series III instrument (Perkin Elmer).

For in vivo experiments, we injected mice intraperitoneally with 150 μg of BODIPY-labeled cholesterol in 50% DMSO plus PBS (added immediately before injection) or vehicle only. After 1 day, we euthanized mice, removed tumors, and weighed each tumor. We dissociated tumors as described for radiotracer studies and used 1 × 10^6^ cells for staining for flow cytometry.

### Mining of scRNA-seq data.

To reanalyze data from Wu et al. (32), we downloaded sequencing files from the Single Cell Portal hosted by the Broad institute at https://singlecell.broadinstitute.org/single_cell/study/SCP1039/a-single-cell-and-spatially-resolved-atlas-of-human-breast-cancers#study-download (brca_mini_atlas_raw_unfiltered.zip). We processed, explored, and visualized the scRNA-seq data set using Cellenics community instance (https://scp.biomage.net/) that is hosted by Biomage (https://biomage.net/). Analysis included reprocessing the data by classifier filter, mitochondrial content filter, GenesVsUMI filter, doublet filter, data integration, and UMAP embedding. Data processing settings can be found in Supplemental Data Sets 1 and 2. The Cellenics web user interface provided further options for data visualization, including UMAP, cluster cell counts, dot plots, and individual gene violin plots.

To analyze data from Martin et al. (22), we used the web user interface from The Broad Institute (https://singlecell.broadinstitute.org/single_cell/study/SCP359/ica-ileum-lamina-propria-immunocytes-sinai?scpbr=immune-cell-atlas#study-visualize), as directed from the Immgen database (https://www.immgen.org/Databrowser19/HumanExpressionData.html). We retrieved data presented in this paper from the web user interface and compared the selected genes between activated T cells and other effector populations. Data can be retrieved and replicated through the above link.

### Statistics.

We performed all data processing in GraphPad Prism v9. Student’s *t* test was used to compare statistical significance between groups of normally distributed samples and MFI, or relative means were analyzed using nonparametric Mann-Whitney tests. A *P* value of less than 0.05 was considered significant, with asterisks (*P < 0.05, **P < 0.01,***P < 0.001, ****P < 0.0001) indicating the degree and magnitude of significance between 2 groups. We created Kaplan-Meier plots and analyzed differences in survival using the the Mantel-Cox test. In assessing statistical differences of tumor burden over time between in vivo groups, we used nonlinear regression for logistical growth to determine significant differences between growth curves. Similarly, for in vitro studies using eFNP-59, nonlinear regression and tests between data set parameters were used to assess significance. A *P*-value cutoff of less than 10^–20^ defined significant differences in expression of specific genes between Louvain clusters. We used analysis of variance (ANOVA) corrected for multiple comparisons to define *P* values for studies with more than 2 experimental groups.

### Study approvals.

The University of Michigan Institutional Animal Care and Use Committee approved all animal procedures under protocol PRO00010534.

### Data availability.

Data are available in public repositories, including scRNA-seq data we acquired from the Broad Institute (https://singlecell.broadinstitute.org/single_cell/study/SCP1039/a-single-cell-and-spatially-resolved-atlas-of-human-breast-cancers#study-download [brca_mini_atlas_raw_unfiltered.zip]) and Immgen (https://www.immgen.org/Databrowser19/HumanExpressionData.html). Additionally, composite data values can be found in the Supporting Data Values XLS file or from the corresponding author upon request.

## Author contributions

GDL, AFB, NGC, and BLV conceptualized this study. NGC and NG analyzed data. NGC wrote the first draft of the manuscript, with editing and input by GDL, BLV, and AFB. NGC, NG, and AFB performed in vitro/ex vivo T cell studies. NGC, AFB, and NG performed all in vivo experiments. NGC performed data query, mining, and reanalysis. NGC, TD, and JS performed all in vivo and in vitro radiation/PET tracer experiments using the laboratory and resources of AFB. NGC, AFB, BLV, PJHS, and GDL contributed to the written discussion and scientific discussions around the manuscript that led to its drafting and formation.

## Supplementary Material

Supplemental data

Supplemental data set

Supporting data values

## Version 1 03/19/2024

In-Press Preview

## Version 2 04/22/2024

Electronic publication
