# Supplementary material for: Evaluating immunotherapeutic outcomes in triple-negative breast cancer with a cholesterol radiotracer in mice
Source: JCI Insight. 2024 Mar 19;9(8):e175320. doi: 10.1172/jci.insight.175320 (PMC11141879; doi:10.1172/jci.insight.175320)
Supplement: Supplemental data [file jciinsight-9-175320-s238.pdf]

Supplemental Figure 1

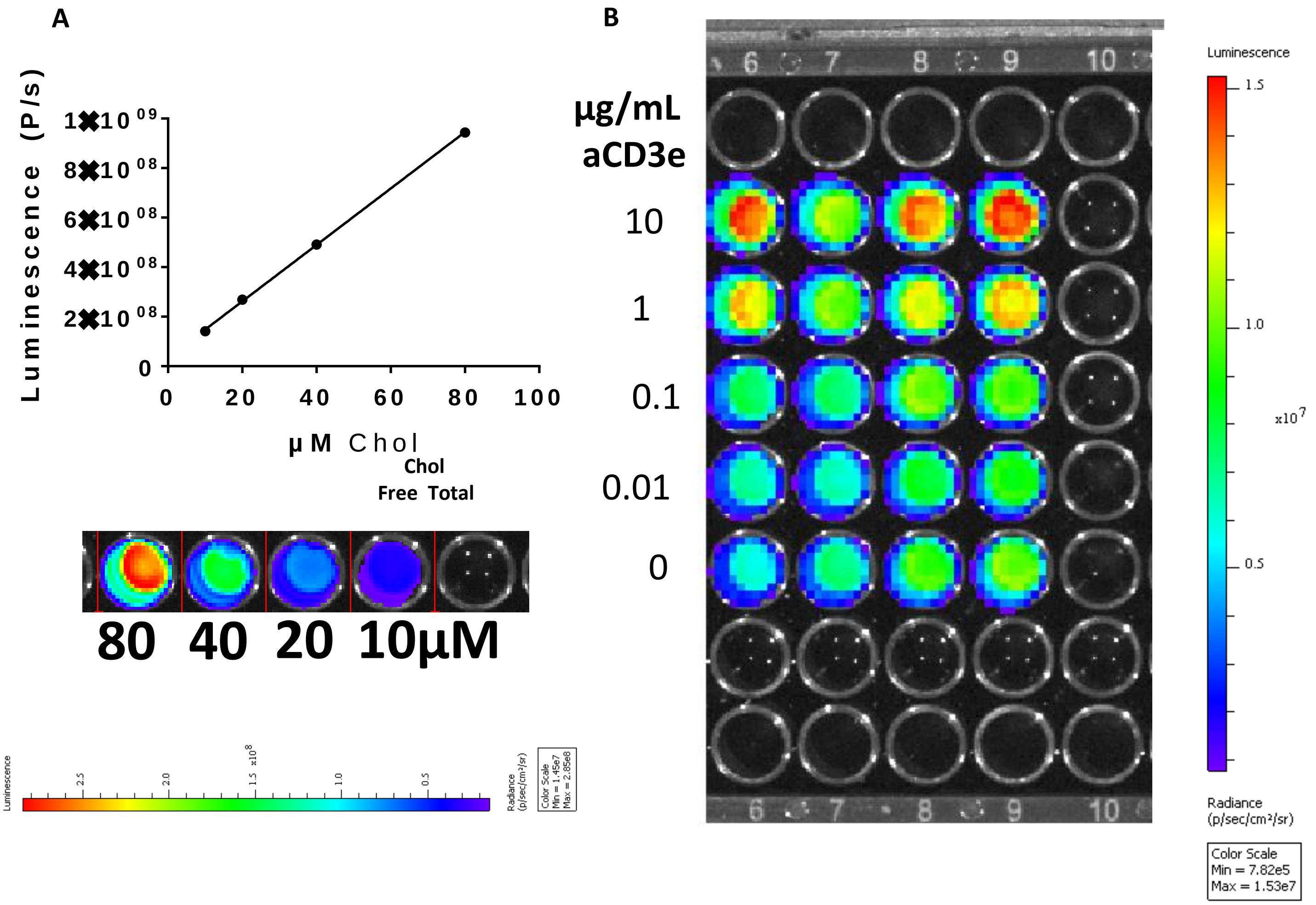

Supplemental Figure 1. A) Standard curve and luminescence image for luminescent cholesterol assay shown in Supplemental figure 2A. B)Plate image after IVIS imaging. Anti-CD3 concentrations were plated decreasing in rows top to bottom as labeled and in duplicate with esterase added on the right (for total cholesterol).

Supplemental Figure 2

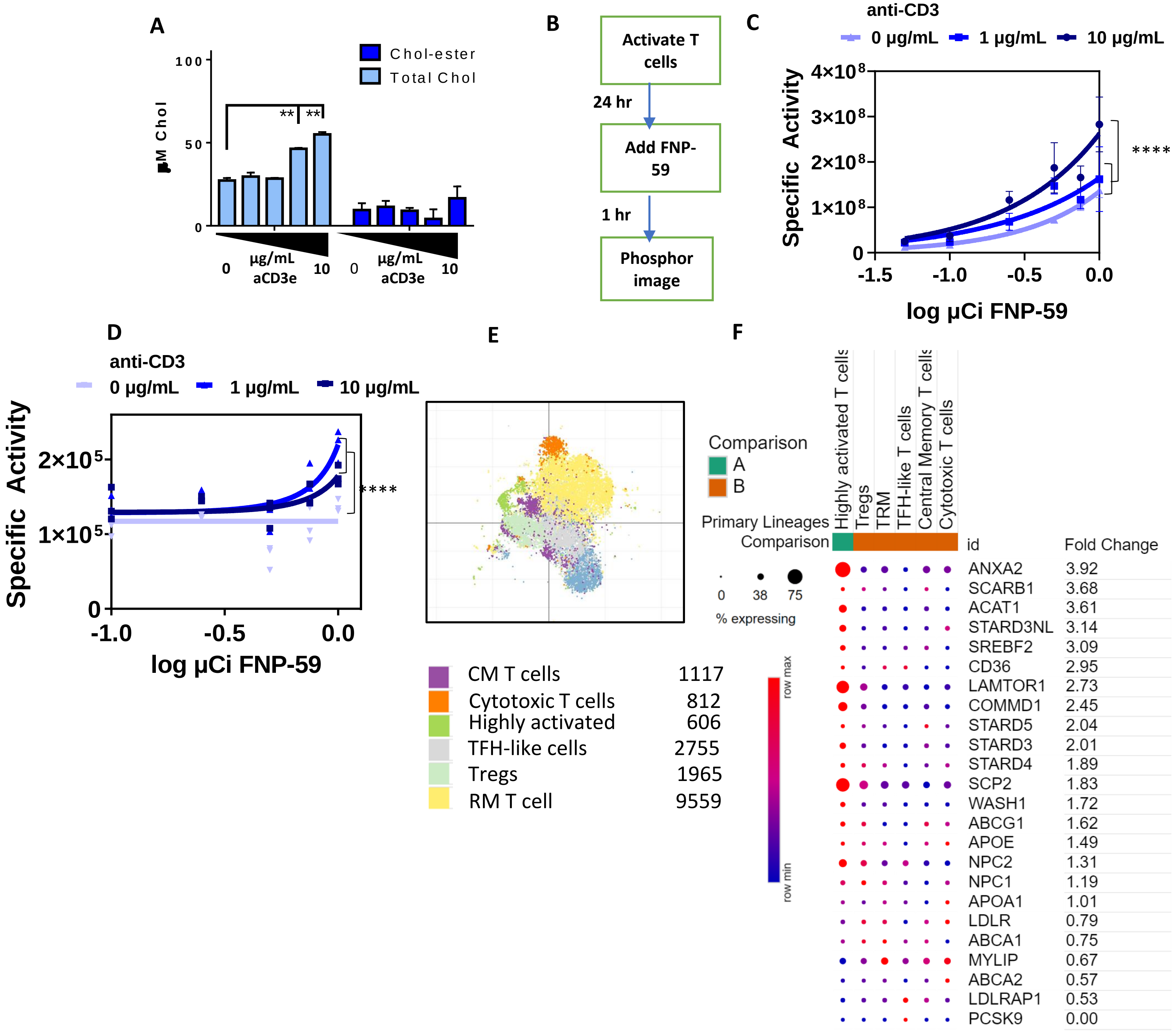

Supplemental Figure 2. Cholesterol analog PET radiotracer, FNP-59, reveals increased uptake of cholesterol in activated T cells. (A) We activated naïve mouse T cells with increasing concentrations of anti-CD3e for 24 hours before quantifying total and esterified cholesterol with a bioluminescence assay. Graphs show mean values + SEM for total cholesterol and cholesterol esters (n=3 per condition). **, p<0.01 by Student's T test. (B) As in the diagram, we activated T cells for 24 hours prior to adding different concentrations of the cholesterol radiotracer analog, FNP-59, for one hour before washing and quantifying cell-associated radioactivity by autoradiography with a phosphor imaging screen. (C) Quantified data for uptake of FNP-59 at different amounts of administered radiotracer. We performed all experiments in triplicate, using comparison of fits with the extra sum-of-squares F test used to compare and test each data set and EC50 against the null hypothesis. Additionally, (D) T cells isolated from healthy human PBMC were activated with human anti-CD3e and treated and analyzed as described for mouse T cells. (E) Using annotated T cell clusters from the Immunological Genome Project's (Immgen) Immune Cell Atlas scRNA-seq data sets on healthy human lamina propria [19], we analyzed genes involved in cholesterol uptake, retention, and trafficking. (F) Panel displays fold change in selected genes related to cholesterol metabolism between the 'highly activated T cell' cluster as compared with all other T cells.

Supplemental Figure 3

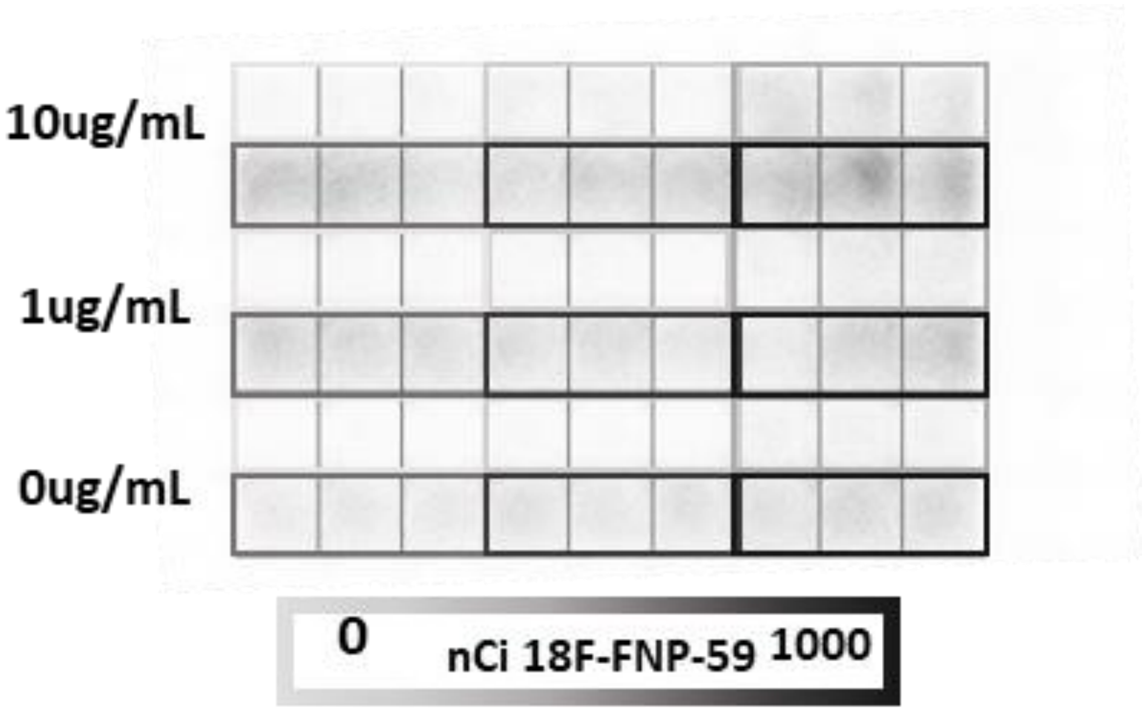

Supplemental Figure 3. Plate image after phosphor screen processing from Figure 4C to show how these data are captured and obtained. FNP-59 concentrations were plated increasing left to right in triplicate. The six concentrations represented by light gray to black color outline boxes were 0.05, 0.1, 0.25, 0.5, 0.75, 1 µCi treatments over 1 hour.

Supplemental Table 1				
Name	P-value	Adj P-value	Odds ratio	Combined score
**Regulation Of Cholesterol Biosynthesis By SREBP (SREBF) R-HSA-1655829**	**7.886e-8**	**9.261e-7**	**8.28**	**135.43**
**Activation Of Gene Expression By SREBF (SREBP) R-HSA-2426168**	**0.000002717**	**0.00002195**	**8.44**	**108.11**

Supplemental Table 1. Increase expression of genes related to cholesterol uptake in cycling T cell populations in patients with triple-negative breast cancer. We re-analyzed major T cell populations from existing single-cell RNA sequencing data [29] using Cellenics software by Biomage (see Figure 6). With Reactome analysis software interface, we compared the "cycling" cluster to all other T cells using Enrichr for pathways ontologically related to cholesterol uptake. Filter cutoffs used $P < 0.05$ and $logFC > 0.1$.

**pAdj values (fig 6D-I)**				**Log FC expression (fig 6D-I)**			
**Cycling vs**	**Rest /mem**	**Transit /eff**	**Dysfunct.**		**Rest /mem**	**Transit /eff**	**Dysfunct.**
ANXA2	1.68E-68	4.68E-48	2.29E-70	ANXA2	0.6526	0.4209	0.5317
SREBF2	3.56E-26	6.74E-34	3.36E-26	SREBF2	0.09925	0.06603	0.05973
LAMTOR1	1.11E-51	3.87E-36	1.10E-20	LAMTOR1	0.40000	0.2548	0.2007
LDLR	1.23E-20	9.92E-37	2.08E-56	LDLR	0.1059	0.09252	0.1155
COMMD1	2.11E-52	1.27E-59	4.43E-55	COMMD1	0.04908	0.1958	0.2055
STARD3NL	4.87E-45	6.40E-46	7.69E-53	STARD3NL	0.2214	0.1502	0.1787
**Rest/mem vs.**		**Transit /eff**	**Dysfunct.**			**Transit /eff**	**Dysfunct**
ANXA2		1.385e-13	1.216e-5	ANXA2		-0.2317	-0.1209
SREBF2		0.001272	0.1003	SREBF2		-0.03322	-0.03952
LAMTOR1		8.771e-9	3.014e-14	LAMTOR1		-0.1441	-0.1983
LDLR		0.1635	0.9657	LDLR		-0.01337	0.009653
COMMD1		6.231e-5	2.402e-4	COMMD1		-0.06401	-0.05425
STARD3NL		2.552e-6	1.165e-3	STARD3NL		-0.07114	-0.04269
**Teff vs.**			**Dysfunct**				**Dysfunct**
ANXA2			6.498e-7	ANXA2			0.1107
SREBF2			1	SREBF2			-0.006304
LAMTOR1			0.002409	LAMTOR1			-0.05415
LDLR			2.394e-5	LDLR			0.02302
COMMD1			0.4722	COMMD1			0.009758
STARD3NL			0.07073	STARD3NL			0.02845

Supplemental Table 2. Adjusted P-values and logFC values of specific genes expressed when comparing between custom clusters from Figure 6. Rest/mem is the annotated resting/memory T cell population, Transit/eff is the transitional/effector T cell population, and Dysfunct is the dysfunctional T cell population.
